# Editorial: Newborn screening for inborn errors of metabolism volume II

**DOI:** 10.3389/fped.2026.1784315

**Published:** 2026-03-10

**Authors:** Mohamed A. Elmonem, Lambertus P. van den Heuvel

**Affiliations:** 1 Department of Clinical and Chemical Pathology, Faculty of Medicine, Cairo University, Cairo, Egypt; 2 Laboratory of Pediatric Nephrology, Department of Development and Regeneration, KU Leuven, Leuven, Belgium; 3 Department of Pediatric Nephrology and Genetics, Radboud UMC, Nijmegen, Netherlands

**Keywords:** exome sequencing, genome sequencing, inborn errors of metabolism (IEM), mass spectrometry, newborn screening (NBS)

Newborn screening (NBS) for rare genetic disorders has been a global cornerstone of preventive medicine for almost 60 years now. So far, it is mainly dependent on the biochemical detection of abnormal metabolites in the blood of apparently healthy newborns. These aberrant metabolites can predict a faulty metabolic pathway that could cause an inherited disorder. The basic concept here is that with the early detection of the abnormal metabolites of a faulty genetic pathway, the disease can be diagnosed before the appearance of the major symptoms and complications and with the proper intervention, the disease morbidity and mortality can be prevented and the affected individual can lead a semi-normal productive life (1). Apart from the clear societal benefits, economic evaluations of running NBS programs in multiple countries have provided solid evidence for the significant health care savings and improved quality of services provided by such programs over the years (2–5).

Wilson and Jungner have provided the first framework of criteria needed for the selection of a genetic disorder to be included in a national NBS program (6). These criteria indicate that the condition should be an important health problem at the screening program location, an accepted treatment modality is available for the disease, facilities for diagnosis and treatment should alsobe available, a recognizable latent or early asymptomatic stage should typically exist, a dependable test is available and is acceptable to target population, the natural history of the disease should be adequately understood and there should be an agreed policy on whom to treat, the cost of case-finding including diagnosis and treatment should be economically balanced in relation to expected expenditure on future medical care, and finally the screening program should be an on-going process and not a limited project (6).

These criteria were initially developed and applied to the biochemically based NBS programs that started with primitive microbial inhibition assays up to current tandem mass spectrometric metabolomic methodologies. Over the last decade, next-generation sequencing (NGS) based technologies, such as exome and genome sequencing, have been established as a second-tier confirmatory testing for conventional first-tier biochemical screening (7–10). More recently, several large-scale pilot studies have reported the advantages of NGS as a first-tier testing procedure and how it could be superior to the standard biochemical NBS approach (11–15) and some argued that biochemical screening should be the second-tier testing choice not the first (14, 15). This could also be a potential solution for the detection of variants of uncertain significance (VUS) in genetic screening next to functional genomics assays.

An NGS-first-tier screening approach is similar to biochemical screening in that it is also highly accurate, reproducible and with high-throughput; however, it can detect virtually all monogenic diseases with or without a measurable metabolic alteration. Although the current establishment and running costs of a genetic newborn screening program are still much higher compared to a biochemical screening programs, genetic testing are getting cheaper every year. Furthermore, unlike biochemical screening, which requires technical and logistic hurdles to overcome to add a new test screening for a new disease, once an NGS screening pipeline is established, introducing a new genetic disease to the initial panel would require minimal technical planning and almost no extra costs (16).

In this genomic era, the decision to select a new disorder to be added to the screening program will be the rate-limiting step not the technical and economic hurdles. For this purpose, the selection criteria developed by Wilson and Jungner have been revisited by Andermann and colleagues to face the future challenges of the new genomic era (17). Their recommendations included the following: the screening program should respond to a recognized societal and medical need, the objectives of screening should be defined at the start of the program, a target population should be strictly defined, strong scientific evidence should exist for theeffectivenessof the screening program, the program should integrate education, testing, clinical services and management, there should be quality assurance, with mechanisms to minimize potential risks of screening, the program should ensure informed choice, confidentiality and respect for autonomy of the population tested, the program should promote equity and access to screening for the entire target population, the program evaluation should be planned from the outset and finally the overall benefits of screening should outweigh the harm (16, 17).

This Research Topic includes four articles applying newborn screening and geneticdiagnosis to the detection of different IEMs. Tan et al., have described two siblings suffering from carnitine palmitoyl transferase II deficiency (CPT II). Unlike most inborn errors of metabolism, which start manifesting within a few months after birth with the accumulation of abnormal metabolites, both siblings described in the report suffered from antenatal cardiomegaly, ventriculomegaly, and polycystic kidneysand both succumbed to their disease within the first two weeks of life.

AlEissa et al., report on the national strategies for screening neural tube defect in Saudi Arabia. They concluded that unlike countries such as Australia and Chile, Saudi Arabia lacks a standardized system for tracking and evaluating neural tube defects outcomes and further efforts need to be directed towards their screening and prevention.

Dang et al., provided the clinical features, diagnosis, treatment, and prognosis of a newborn with isolated adrenocorticotropic hormone deficiencycaused by variants in the *TBX19* gene. They further performed a literature search and summarized the phenotype of 34 other probands. The collective main clinical manifestations of the syndrome were hypoglycemia, jaundice, convulsions, feeding difficulties, poor mental response, hypotonia, and growth retardation.

Kononets et al., have revised the status of tandem mass spectrometry and its roles in newborn screening through a systematic analysis of the published literature about the topic. They identified future areas of interest in the subject area to be: “selective screening for IEM,” “new treatments for IEM,” “new disorders considered for MS/MS testing,” “ethical issues associated with newborn screening,” “new technologies that may be used for newborn screening,” and “use of a combination of MS/MS and gene sequencing”.

In conclusion, NGS based newborn screening technologies have many advantages over biochemical based screening. Although the proper scale of obstacles and shortcomings of this approach will not be clear except with its application at the national or multinational levels, we seem to be getting there very quick.
